# High-capacity data hiding for medical images based on the mask-RCNN model

**DOI:** 10.1038/s41598-024-55639-9

**Published:** 2024-03-26

**Authors:** Hadjer Saidi, Okba Tibermacine, Ahmed Elhadad

**Affiliations:** 1 Department of Computer Science, University of Biskra, Biskra, Algeria; 2 National School of Artificial Intelligence, Algiers, Algeria; 3 Department of Computer Science, Faculty of Computers and Information, South Valley University, Qena, Egypt

**Keywords:** Medical image security, Data hiding techniques, Mask-RCNN, Deep learning in medical imaging, High-capacity embedding, DCT-based steganography, Image processing, Machine learning, Software

## Abstract

This study introduces a novel approach for integrating sensitive patient information within medical images with minimal impact on their diagnostic quality. Utilizing the mask region-based convolutional neural network for identifying regions of minimal medical significance, the method embeds information using discrete cosine transform-based steganography. The focus is on embedding within “insignificant areas”, determined by deep learning models, to ensure image quality and confidentiality are maintained. The methodology comprises three main steps: neural network training for area identification, an embedding process for data concealment, and an extraction process for retrieving embedded information. Experimental evaluations on the CHAOS dataset demonstrate the method’s effectiveness, with the model achieving an average intersection over union score of 0.9146, indicating accurate segmentation. Imperceptibility metrics, including peak signal-to-noise ratio, were employed to assess the quality of stego images, with results showing high capacity embedding with minimal distortion. Furthermore, the embedding capacity and payload analysis reveal the method’s high capacity for data concealment. The proposed method outperforms existing techniques by offering superior image quality, as evidenced by higher peak signal-to-noise ratio values, and efficient concealment capacity, making it a promising solution for secure medical image handling.

## Introduction

The medical information of patients must be protected from unauthorized access. Generally, medical information security refers to the rules of security policies that strictly ethical rights and privacy of the patient and must be concerned by entities. There are many widely used standards and tools for protecting personal medical information such as the ISO27799 (Security Management in Health Using ISO/IEC/27799)^1^, Cryptography^2^, and Steganography^3^ techniques. ISO27799 is a standard that provides security management guidelines for health organizations to protect medical information. Cryptography is data protection using an encryption provider which converts medical information into unintelligible text using a secure key. Steganography can be defined as the use of a host (container) data to hide or embed a piece of information that is hidden directly in media content, in such a way that it is imperceptible to a human observer but can be detected/extracted easily with a computer^4^. Steganography is employed in various useful applications, such as in Medical Imaging Systems where a separation is considered necessary between patients’ medical files and their personal information for the sake of confidentiality^5^.

Medical image steganography aims at delivering a modified medical image to secure the transfer of hidden information inside with little perception of third-party. Medical image steganography is an emerging field of research that aims to embed secret information into medical images in a way that is imperceptible to the human eye, while ensuring the integrity and confidentiality of the medical data. The primary motivation behind medical image steganography is to protect patient privacy by preventing unauthorized access to sensitive medical information. High-capacity data hiding techniques, such as deep learning based models, have been developed to address the limitations of traditional steganography methods, which have limited embedding capacity and are vulnerable to attacks. The RCNN-Mask model is a deep learning-based approach that can effectively embed a large amount of secret data into medical images, while maintaining the visual quality of the images and ensuring robustness against attacks.

Deep learning has emerged as a powerful tool for data hiding approaches, enabling the development of high-capacity, robust and imperceptible steganography techniques. Deep learning-based approaches leverage the power of neural networks to learn the optimal mapping between cover images and secret data, allowing for the generation of stego-images that are visually indistinguishable from their cover counterparts. These techniques are designed to embed secret data into the cover images in a way that is robust against attacks, such as image processing or compression, while maintaining the quality of the stego-images. Furthermore, deep learning-based approaches can be adapted to different types of data, including images, audio, and video, allowing for the development of versatile and flexible steganography techniques. Overall, deep learning-based data hiding approaches have revolutionized the field of steganography, enabling the development of advanced and effective techniques for data protection and privacy.

The proposed high-capacity data hiding for medical images based on the RCNN-mask model is a novel steganography technique that aims to embed a large amount of secret data into medical images while maintaining their visual quality and ensuring robustness against attacks. The RCNN-mask model, which is a deep learning-based object detection and segmentation model, is used to identify and segment regions of interest in the cover image, which are then used to embed the secret data. The proposed technique leverages the high-capacity of the RCNN-mask model to embed a large amount of secret data into the cover image, while ensuring that the stego-image remains imperceptible to the human eye. Experimental results show that the proposed technique achieves high embedding capacity and low distortion, as well as robustness against various attacks, such as JPEG compression and cropping. Overall, the proposed high-capacity data hiding for medical images based on the RCNN-mask model is a promising steganography technique that can be used to protect sensitive medical data while ensuring its confidentiality and integrity.

The advent of digital imaging in medical diagnostics has revolutionized healthcare, enabling the storage, sharing, and detailed analysis of medical images. However, this digital transition also introduces significant challenges in ensuring the security and privacy of sensitive patient information embedded within these images. Existing methods for data hiding within medical images often struggle to achieve a harmonious balance between embedding capacity, image integrity, and robustness against attacks. Specifically, traditional techniques tend to compromise on visual quality to increase payload capacity or fail to adequately protect embedded data against common image processing attacks, such as noise addition or compression. Furthermore, many current approaches lack the sophistication to selectively embed information in regions that do not compromise the diagnostic value of medical images, leading to potential risks in clinical interpretation. These technical gaps underscore the need for an advanced data hiding method that not only supports high-capacity embedding without degrading image quality but also ensures the robust protection of embedded data, all while preserving the diagnostic usability of medical images. Addressing these challenges requires a nuanced understanding of both medical image processing and security principles, guiding the development of a method that adeptly navigates the trade-offs inherent in secure medical data handling.

In the era of digital healthcare, the significance of medical images has grown exponentially as essential components of patient records and medical research data. Recent reports highlight a substantial increase in the utilization of digital medical images across various healthcare settings, underscoring their critical role in enhancing diagnostic accuracy, facilitating telemedicine, and supporting advanced medical research. However, this reliance on digital imaging also brings to the fore concerns regarding the security and privacy of sensitive patient information. Studies such as “HIDEmarks: Hiding multiple marks for robust medical data sharing using IWT-LSB”^6^ and “Robust copyright protection technique with high-embedding capacity for color images”^7^ have shed light on innovative approaches to protect this data, yet gaps remain in ensuring these methods meet the nuanced demands of medical imaging. These works emphasize the urgent need for robust, high-capacity data hiding techniques that can safeguard patient information without compromising the diagnostic integrity of medical images. Motivated by these challenges, our work aims to bridge these gaps by introducing a sophisticated data hiding method tailored for the unique requirements of medical imaging. By leveraging the latest advancements in deep learning and steganography, we strive to offer a solution that upholds the confidentiality of embedded data while ensuring the visual and diagnostic quality of medical images remains intact.

The motivation behind this work is to address the growing need for robust data security in medical imaging without compromising the diagnostic value of medical images. As medical imaging plays a crucial role in diagnosis and treatment planning, ensuring the confidentiality and integrity of patient data embedded within these images is paramount. As medical images are shared and stored electronically, the risk of unauthorized access and potential misuse of sensitive patient data escalates. Our work aims to address this challenge by providing a secure method for embedding patient information directly into medical images without impairing their diagnostic value. The key contributions of our study are summarized as follows:

- We introduce a novel data hiding technique based on the mask region-based convolutional neural network (Mask-RCNN) for identifying medically insignificant regions within DICOM images for secure data embedding.
- The proposed method utilizes discrete cosine transform (DCT)-based steganography to embed patient information discreetly, ensuring the integrity and diagnostic quality of medical images are maintained.
- Our approach is demonstrated to achieve high embedding capacity and maintain image quality through rigorous evaluation on DICOM images, outperforming existing methods in terms of robustness, payload capacity, and image fidelity.

The manuscript is organized as follows: “Related work” provides a detailed review of related work, highlighting the gap our study aims to fill. “The proposed method” describes the methodology, including the use of Mask-RCNN for region identification and the DCT-based steganography technique for data embedding. “Experiments” presents the experimental setup, including the dataset used, evaluation metrics, and comparative analysis with existing methods. “Robustness analysis” discusses the results, emphasizing the efficacy and benefits of our approach. Finally, “Conclusion” concludes the paper with a summary of our findings and potential directions for future research.

## Related work

Steganography’s significance is rooted in its ability to conceal the presence or absence of concealed information from unintended recipients, in stark contrast to cryptography, where decryption alone reveals the message. In this section, we delve into pivotal endeavors concerning safeguarding medical data through steganography, while also exploring contemporary methodologies integrating deep learning for steganographic applications over recent years.

### Medical image steganography

In^8^, Bozhidar et al. presented an innovative steganography method termed BOOST, designed to conceal user data within medical images. Their approach unfolded in two distinct stages: Initially, the confidential patient data underwent encryption using a novel “pseudorandom generator based on the nuclear spin generator” technique, resulting in encrypted data. This encrypted output was subsequently transcribed into a binary sequence using an ASCII table. In the subsequent step, this binary sequence found its place within the least significant bit of the non-black pixels in the image. Notably, their method achieved remarkable results, boasting PSNR values surpassing 113 dB, all while accommodating a payload capacity of 0.74 bits per pixel. The substantial payload capacity emphasizes the potential for real-world applications. However, it is important to consider the computational overhead of these encryption and embedding processes, especially when dealing with large medical image datasets.

In^9^, Romany et al. introduced an encompassing steganography method that amalgamates several techniques for robust data hiding within medical images. They proposed the application of RSA encryption for safeguarding sensitive information, the Ripplet Transform for image manipulation, and LSB substitution for embedding secret data. An adaptive genetic-algorithm-based optimum pixel adjustment process (OPAP) was implemented to enhance imperceptibility by fine-tuning the stego image. This comprehensive approach demonstrated resilience against RS attacks and established that Discrete Ripplet Transform (DRT) yielded superior results in comparison to Integer Wavelet Transform (IWT). Notably, the achieved PSNR values ranged from 49 to 56 dB, indicating a trade-off between visual quality and payload capacity.

In^10^, Songul and Engin presented an innovative steganography technique termed “Genetic Algorithm-Optimum Pixel Similarity”. This approach leverages pixel similarity and LSB embedding to seamlessly integrate a substantial amount of data, specifically 10,000 characters, into 256 $\times$ 256 medical images. What sets this method apart is its ability to achieve embedding without resorting to data compression techniques. The fitness function for the genetic algorithm is adopted from PSNR, with random selection as the key method. Impressively, the average PSNR achieved was recorded at 47.41 dB, highlighting the delicate balance between imperceptibility and embedding capacity.

Partha et al. explored patient data protection in^11^ through a novel steganographic method, employing support vector machine (SVM) and discrete wavelet transform (DWT). The SVM was utilized for the recognition of regions of interest (ROI) and non-ROI (NROI) within medical images. RGB components were subjected to IWT, and a circular array technique facilitated the integration of confidential information within NROI pixels. Impressively, this approach yielded an average PSNR value of 64 dB, showcasing its potential for robust and secure patient data embedding. In another study^12^, a robust and reversible data hiding scheme was proposed, involving a support vector neural network (SVNN) classifier and the contourlet transform method. The SVNN was trained to identify suitable pixels for concealment, with the HL band of the CT coefficient serving as the container for hidden data. The method was rigorously analyzed with and without noise, demonstrating exceptional results with a PSNR value of up to 89.3253 dB, outperforming the SVNN-wavelet approach from^13^.

In^14^, an innovative approach was introduced that encoded patient data using enhanced Huffman compression coding for enhanced payload capacity and security. This encoded data was then concealed within medical images using pixels contrast (PC) and the Henon map algorithm. The study evaluated outcomes based on histogram analysis, PSNR, and SIMM metrics, with achieved PSNR values ranging between 70 and 71 dB. A novel steganography technique was proposed in^15^, utilizing a combination of a 3-D chaotic system, one-particle quantum walk (QW), and particle swarm optimization (PSO). This intricate methodology ensured the privacy of medical data by generating sequences for PSO through chaotic systems and QW, which were then utilized to replace confidential medical images with concealed data. Despite its high visual quality, this technique achieved an average PSNR of 44.1 dB, reflecting inherent limitations in data capacity. In^16^, the authors introduced a technique for securely compressing 2D medical images, such as MRI, CT, and ultrasound scans, to efficiently manage storage space while maintaining image integrity and privacy. The method employs a multi-level compression strategy using a dictionary mechanism, combined with a 256-bit symmetric key encryption based on a hashing technique to ensure data security. Additionally, the fuzzy trapezoidal correlation method is utilized for accurately reconstructing the original image from its compressed state, ensuring minimal quality loss. The approach has demonstrated significant reductions in image size (up to 58).

The steganography technique presented by Hashim et al. in^17^ targeted data security during transmission within an IoT framework. Encrypted patient data was divided into blocks and concealed within medical images using the Henon map parameters for random pixel selection. This technique demonstrates effective use of steganography mechanisms for IoT data security. Prasanth et al. introduced an invisible watermarking scheme in^18^ for embedding patient information into EGG signals for telemedicine applications. A QR code of patient data was decomposed and utilized for watermarking EGG signals. This intricate approach provided a unique approach to securing medical information within telemedicine applications.

Arunkumar et al. proposed a novel technique in^19^ for secure medical image transmission. The medical image was encrypted using the logistic chaotic map, followed by embedding using an embedding distortion measure based co-accurate matrix. The method prioritized security and yielded high visual quality while ensuring secure image transmission. The secure steganography method outlined in^20^ incorporated a shell matrix and LSB for enhanced data security. While the method exhibited high payload capacity, it required substantial computational resources for high-resolution images, thereby impacting complexity. The authors, in^21^, introduced a novel approach involving a genetic algorithm to enhance PSNR levels in Stego images. The technique aimed to cover a medical image with a natural image, utilizing a combination of mechanisms such as one-point crossover, random resetting mutation, and tournament selection. While the method achieved infinite PSNR and SSIM values without causing distortion, its complexity remained a significant consideration.

Research in the field of medical image data hiding has laid a solid foundation but has also encountered several challenges that our study aims to address. Notably, existing techniques often struggle with balancing the trade-off between embedding capacity, robustness against attacks, and preserving image quality. To mitigate this challenge, our proposed approach leverages advanced deep learning architectures, such as Mask-RCNN, to precisely identify regions within medical images suitable for data embedding while minimizing the risk of diagnostic information loss. Moreover, we employ sophisticated embedding techniques, such as the discrete cosine transform (DCT), to embed data in frequency components least perceptible to the human eye, thereby ensuring both robustness and imperceptibility. Additionally, our model incorporates adaptive selection mechanisms to intelligently prioritize embedding in regions with optimal texture and complexity characteristics, further enhancing the security and reliability of the embedding process. By addressing these key challenges, our approach represents a significant advancement in the field, offering a more balanced and effective solution for high-capacity data hiding in medical images.

## The proposed method

### Overview

The proposed model innovatively integrates the mask region-based convolutional neural network (Mask-RCNN) with discrete cosine transform (DCT) for high-capacity data hiding within medical images. At its core, the model employs Mask-RCNN, a state-of-the-art deep learning framework known for its precision in instance segmentation tasks. This model is adept at identifying and segmenting regions within medical images that are medically less significant, thereby earmarking them as potential areas for secure data embedding. The choice of Mask-RCNN is motivated by its dual capability to classify individual pixels in an image while precisely delineating the boundaries of objects, making it an ideal candidate for isolating regions where embedding can occur without affecting the diagnostic value of the image.

Once these regions are identified, the model utilizes the discrete cosine transform (DCT), a cornerstone technique in signal processing that transforms spatial domain data into frequency domain. This transformation facilitates the embedding of sensitive information into the frequency components of the image, specifically targeting the mid-frequency components. This choice is strategic; the human eye is less sensitive to changes in these components, ensuring that the embedding remains imperceptible. Moreover, DCT provides a mechanism to adjust the embedding intensity, allowing for a flexible trade-off between embedding capacity and image quality.

The embedding process is further refined through an adaptive selection mechanism, which evaluates the suitability of identified regions based on their texture and complexity, ensuring that data is embedded in areas where it is least likely to be detected or affect image quality. This nuanced approach, combining Mask-RCNN’s segmentation prowess with DCT’s embedding efficiency, represents a significant technical advancement in the field of medical image security.

**Figure 1:**
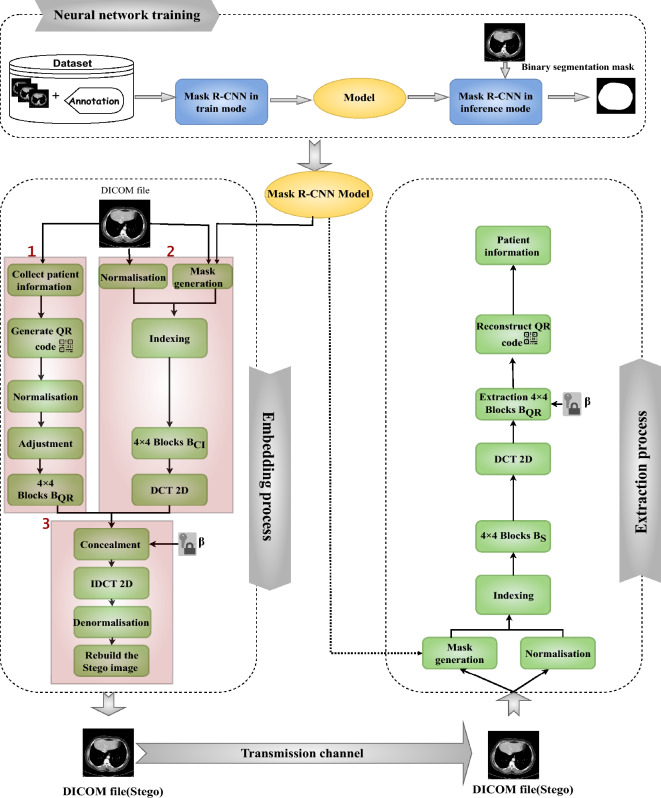
Overview of the proposed steganography method.

This study’s contribution lies in the creation of DICOM files that seamlessly integrate patient information into medical images with an exceedingly minimal impact-almost inconsequential-in order to safeguard against misdiagnosis, all achieved through the application of steganography principles. The devised approach involves concealing patient data within areas of the medical image that hold marginal relevance. Here, “insignificant areas” refer to regions devoid of crucial medical data, such as the black segments found in grayscale DICOM images. The identification of these areas is facilitated by deep learning (DL) models, which effectively discern non-essential regions within the original images. Subsequently, sensitive medical information is discreetly embedded within these inconspicuous regions using DCT-based steganography. A comprehensive visual representation of the proposed methodology is depicted in Fig. 1, outlining three fundamental stages: neural network training, embedding, and extraction.

### Neural network training

**Figure 2:**
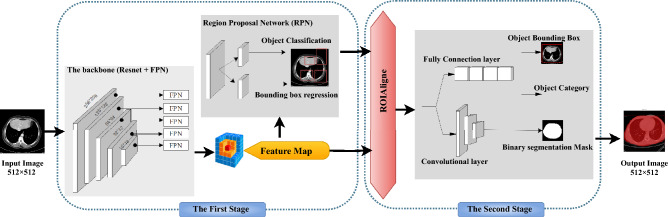
Mask R-CNN architecture.

The key-concept in the proposed method is the detection of insignificant areas in medical DICOM images which will be exploited to conceal sensitive information. We assume that the best way to detect these regions is by correctly detecting the main objects in the image. In the literature, CNN-based methods outperform traditional techniques in the detection and segmentation of objects inside images. Thus, we adopt Mask-RCNN architecture^22^, as one of the efficient techniques especially in the field of medical images. This architecture is proposed to detect the main objects which represent the significant area that should be kept safe during information embedding. To train the Mask-RCNN model to obtain binary segmentation masks, we use the architecture depicted in Fig. 2 on various DICOM files datasets. This architecture is divided into two stages:

1. First stage: it takes an image and produces a feature map and regions proposals. The feature map is obtained through a series of operations conducted on the original image by a backbone constructed from CNN layers (ResNet and feature pyramid). Region proposal network takes the feature map and produces regions that may contain objects.
2. Second stage: it consists of aligning regions of interests (RoIalign). It takes as input feature map and region proposals and generates as output the fixed size regions of interest from region proposals, and three parallel branches for predicting: object category, instance bounding-box, and binary segmentation masks.

This architecture is trained on various datasets to determine the binary segmentation mask where 1’s represents object pixels (significant region) and 0’s represents the background of the image (i.e. the insignificant area). The loss function used to train this model is defined by Eq. (1).

1 $$\begin{aligned} L = L_{class}+L_{box}+L_{mask} \end{aligned}$$

where $L_{class}$ is the classification loss, $L_{box}$ is the bounding box regression loss, and $L_{mask}$ is the mask Loss.

### Embedding process

The process of embedding, also known as the concealment process, encompasses a series of sequential steps aimed at seamlessly integrating confidential patient-related data into DICOM images, ultimately giving rise to a Stego image. To elaborate, the focal point of this embedding is the inconspicuous area previously identified using the Mask-RCNN model. This entire procedure can be delineated into three distinct phases, outlined in the subsequent subsections.

#### Sensitive information preprocessing

**Figure 3:**
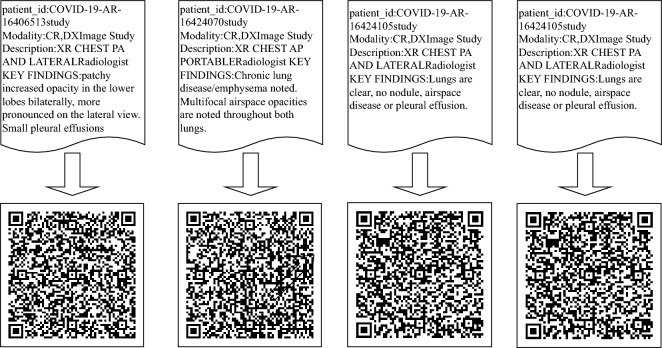
Examples of patient data with their corresponding QR codes.

In this first phase, the sensitive patient information is retrieved from the DICOM file and transformed into a QR code image via QR Code generator. Figure 3 depicts some examples of patient information with their corresponding QR codes generated using the Zxing library^23^.

The Generated QR code image is then normalized using Eq. (2) to convert the values within the image to a range between 0 and 1. After that, the normalized image is adjusted using Eq. (3), where the value of $\alpha$ is fixed experimentally ($\alpha$ = 0.02). The adjusted image is then divided into 4 $\times$ 4 mini matrices called blocks ($B_{Qr}$) that are hidden one by one during the concealment phase.

2 $$\begin{aligned} Norm( {\text {Img}})= & {} \frac{{\text {Img}}-{\text {Min}}({\text {Img}})}{\max ({\text {Img}})-{\text {Min}}({\text {Img}})} \end{aligned}$$

3 $$\begin{aligned} Adjustment (M s g)= & {} \left\{ \begin{array}{c}1-\alpha , M s g \ge 1 \\ \alpha , M s g=0\end{array}\right. \end{aligned}$$

#### Cover preprocessing

The cover, derived from the DICOM file, is subjected to a dual stage preprocessing procedure. Initially, the mask-RCNN model is employed in inference mode to pinpoint the inconsequential regions within the cover, which yields a binary segmentation mask. Subsequently, given that DICOM images are frequently encoded in 16-bit grayscale, the cover is normalized using Eq. (2) to effectively remap its values within the [0, 1] interval.

The resulting mask obtained from the first preprocessing step is utilized to determine the indices corresponding to the insignificant areas within the normalized cover image, referred to us as ($C_{ins}$) image. These insignificant areas are subsequently divided into blocks with dimensions of 4 $\times$ 4, denoted as ($B_{Cins}$). The transformation coefficients of ($B_{Cins}$) are then computed using the two-dimensional discrete cosine transform function (2D-DCT)^24^, resulting in the generation of ($B_{DCins}$) blocks. The 2D-DCT for a matrix I (with dimensions M $\times$ N) is calculated using the formula specified in Eq. (4)

4 $$\begin{aligned} C(u,v)= \alpha (u).\alpha (v) \times \left[ \sum _{m=0}^{M-1} \sum _{n=0}^{N-1} I(m,n) \times cos \frac{ (2m+1)u\pi }{ 2M } cos \frac{ (2n+1)v\pi }{ 2N } \right] \begin{array}{c} 0 \le u\le M-1 \\ 0 \le v \le N-1 \end{array} \end{aligned}$$

where $$ a(u)= \left\{ \begin{array}{c} 0 \le u\le M-1 \\ 0 \le v \le N-1 \end{array}\right. $$

(*m*, *n*) and *I*(*m*, *n*) correspond to the position values and the pixel value at position (*m*, *n*) in the spatial domain respectively. *C*(*u*, *v*) isthe corresponding position value and the frequency coefficient at position (*u*, *v*) in the transform domain.

#### Information concealment

During this stage, the 4 $\times$ 4 blocks originating from the cover, denoted as ($B_{DCins}$), and the 4 $\times$ 4 blocks representing the secret message, referred to as ($B_{Qr}$) and generated in the prior phase, are merged together-specifically, ($B_{Qr}$) is concealed within ($B_{DCins}$)-resulting in the formation of the corresponding block ($B_s$) within the Stego image. This concealment process is executed through the application of Eq. (5).

5 $$\begin{aligned} B_S = IDCT2 (Qun(B_{DCins},B_{Qr})) \end{aligned}$$

where

- $B_s$: Represents the block of the Stego image resulting from the concealment process.
- IDCT2 is the inverse two-dimensional discrete cosine transform function (2D-IDCT). This function is explained by Eq. (6).
  6 $$\begin{aligned} I(m,n)= \alpha (u).\alpha (v) \times \left[ \sum _{u=0}^{M-1} \sum _{v=0}^{N-1} C(u,v)\times cos \frac{ (2m+1)u\pi }{ 2M } cos \frac{ (2n+1)v\pi }{ 2N } \right] \end{aligned}$$
- *Qun*: Denotes the function that integrates the $B_{Qr}$ block into $B_{DCins}$ and generates the pre-Stego block$B_{SQ}$. This block is computed using the formula depicted in Eq. (7).
  7 $$\begin{aligned} B^{i,j}_{SQ} = k + \left( \frac{4}{\beta } \times B^{i,j}_{Qr}\right) \quad ; \qquad \left( \frac{4k}{\beta }\right)< \mid B^{i,j}_{DCins}\mid < \frac{(4k+1)}{\beta } \end{aligned}$$
  where:
  - i,j: are respectively the i-th and j-th ligne and column in the block.
  - $\beta$: is the number of intervals that satisfy the cover coefficients on the interval of [0, 4].
  - $$k \in {1,2,3,\ldots , \beta -1}$$.

**Figure 4:**
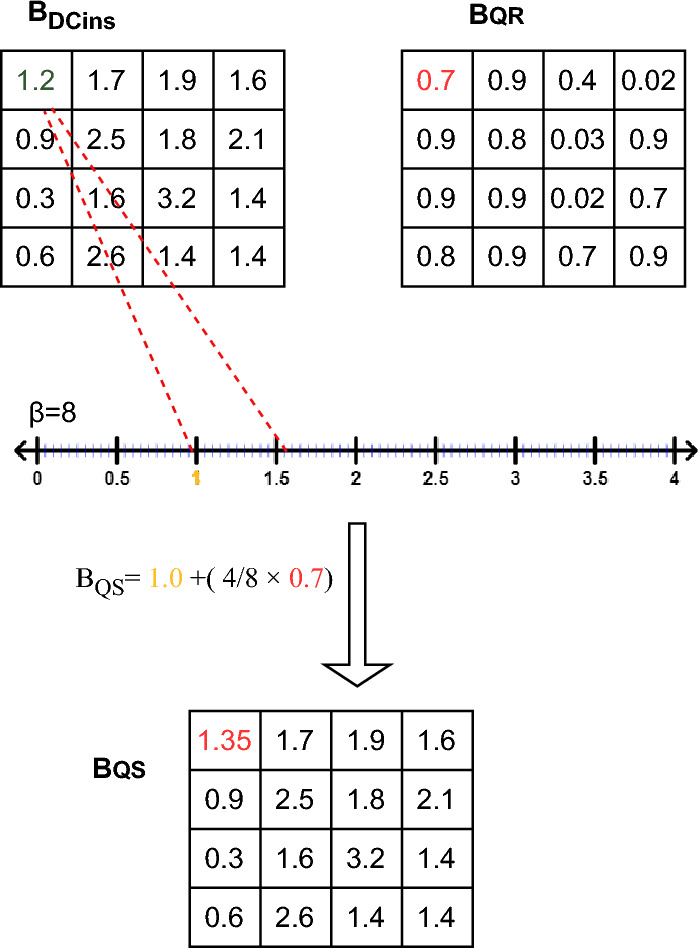
A visual illustration of the hiding technique when $\beta$ = 8.

Subsequently, the inverse two-dimensional discrete cosine transform function (2D-IDCT) is employed on $B_{SQ}$, yielding the ultimate $B_S$ that characterizes the Stego blocks. Following this, the Stego blocks are amalgamated to forge the Stego image, which subsequently undergoes de-normalization to confine values within the [0, 65535] range. This critical step guarantees that the Stego image adheres to the 16-bit DICOM file format, conserving the initial encoding scheme. An illustrative demonstration of the concealment process can be found in Fig. 4. Algorithm 1 prescribes the sequential steps that constitute the embedding process.

**Algorithm 1:**
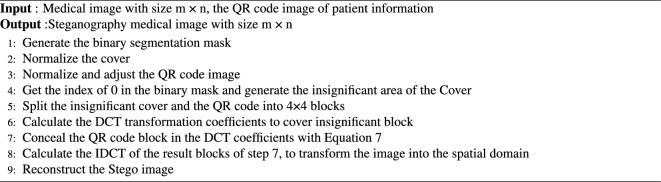
The embedding algorithm.

### Extraction process

The extraction process serves as the reverse of the concealment process, with the aim of recovering the patient data hidden within Stego images. This retrieval is exclusively authorized for users possessing the requisite key. The extraction unfolds across two distinct phases: mask generation and the extraction process.

In the mask generation phase, the Stego image is subjected to the Mask R-CNN model operating in inference mode. This operation generates a binary segmentation mask tailored to pinpoint insignificant regions. This mask, a critical tool, facilitates the identification and indexing of these areas-precisely where the secret data has been concealed.

Moving to the extraction process, an essential preliminary step involves normalizing the Stego image to ensure pixel values are confined within the [0, 1] range. Leveraging the binary mask associated with the Stego image, the positioning of pixels utilized for concealment is discerned. This determination is pivotal in the creation of the insignificant Stego matrix. Subsequently, the matrix is partitioned into $4\times 4$ blocks and subjected to the Discrete Cosine Transform (DCT) function, transitioning it into the frequency domain. The outcome is a set of transformation coefficients. By employing the inverse merge operation (*IQun*), these coefficients facilitate the extraction of the mini block corresponding to the QR code, denoted as $B_QR$. The extraction equation is formally expressed as detailed in Eq. (8)

8 $$\begin{aligned} B_{QR} = IQun(DCT2(B_S)) \end{aligned}$$

where the inverse merge equation (IQun) is calculated by Eq. (9).

9 $$\begin{aligned} B_{QR}=(B_S-k)\times \beta /4 ; \qquad \left( \frac{4k}{\beta }\right)< \mid B^{i,j}_{S}\mid < \frac{(4k+1)}{\beta } \end{aligned}$$

Finally, the resulting $B_Q$ blocks are then concatenated to produce the QR code of the patient information. The reverse process is summarized by Algorithm 2

**Algorithm 2:**
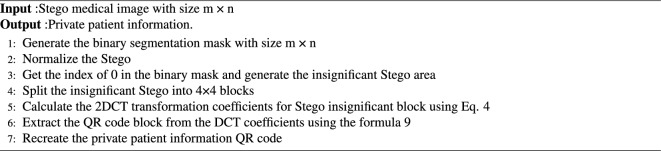
The extraction algorithm.

## Experiments

### CHAOS dataset

CHAOS dataset aims to segment abdominal organs (liver, kidneys, and Spleen) using CT and MRI data^25^. It consists of two datasets, Each one corresponding to a series of DICOM images. The first comprises CT images of 40 different patients with a healthy liver. The patient orientation and alignment are the same for all the data sets. The data consists of 16-bit DICOM images with a resolution of $512\times 512$, an x–y spacing of 0.7–0.8 mm, and an inter-slice distance (ISD) of 3–3.2 mm. The second database includes 120 DICOM data sets from two different MRI sequences, each of which is being routinely performed to scan the abdomen using different radiofrequency pulse and gradient combinations. The data sets are acquired by a 1.5 T Philips MRI, which produces 12 bit DICOM images having a resolution of $256 \times 256$. The ISDs vary between 5.5 and 9 mm (average 7.84 mm), x–y spacing is between 1.36 and 1.89 mm (average 1.61 mm) and the number of slices is between 26 and 50. we have randomly selected 1200 DICOM images from the CHAOS dataset. These images are divided into 1023 images (11 patient images) for training, 199 images for validation (3 patient images) 10 images for testing. We manually created the annotation of these images using VIA Annotation Software^26^, with the intention to make a semantic segmentation to separate the foreground that we consider as Significant area from the background that we consider as insignificant area.

### Mask RCNN model training and evaluation

We leveraged transfer learning to prepare a Mask-RCNN model that detects insignificant areas in DICOM images. We started by fine-tuning the pre-trained weights of the MS COCO model^27^, which is a large object detection and instance segmentation dataset that comprises 328k images with 91 labeled categories. To fine-tune this model, we used the implementation of MASK-RCNN proposed by Matterport in^28^ and we started the training on the CHAOS dataset with the MS COCO weights to produce a variation of the network that targets our detection goals.

**Table 1:** Mask R-CNN configuration.

Parameter	Value
Backbone	resnet101
BATCH_SIZE	4
FPN_CLASSIF_FC_LAYERS_SIZE	1024
PU_COUNT	1
IMAGES_PER_GPU	4
LEARNING_MOMENTUM	0.9
LEARNING_RATE	0.001
RPN_TRAIN_ANCHORS_PER_IMAG	256
STEPS_PER_EPOCH	10
VALIDATION_STEPS	50
WEIGHT_DECAY	0.0001

Table 1 presents the configuration details for training our variant of the Mask-RCNN model. The parameters listed in the table include *the backbone architecture*, which is the ResNet101 architecture in this case. The *batch size*, which is the number of images used in each training iteration, is set to 4. The *Feature Pyramid Network (FPN)* used for classification is a fully connected layer with a size of 1024. The *learning rate* and *momentum parameters* are set to 0.001 and 0.9 respectively. And to prevent overfitting, we set the *weight decay* parameter to 0.0001. The *RPN Train Anchors per Image* parameter, which refers to the number of anchors used in the region proposal network (RPN) during training, is set to 256. and the *Images Per GPU* parameter is set to 4, indicating that each GPU processes 4 images at a time. The *Steps Per Epoch* parameter is set to 10, and the *Validation Steps* parameter is set to 50. These parameters control the training process and the number of training and validation iterations.

**Figure 5:**
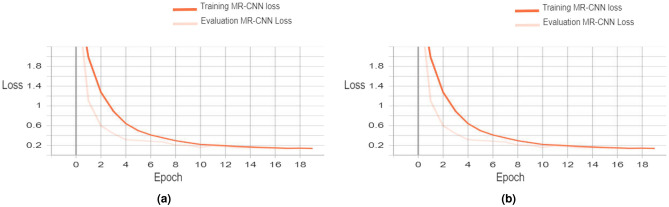
Model training and validation losses.

The training was conducted on a machine empowered by Core I7 and 10th generation processor, Intel UHD graphics, and 16 GB of RAM. The training is done in 28 epochs (8 epochs for the head and 20 epochs to fine-tune all layers). The histograms of the training and validation losses are presented in Fig. 5. Sub-figure (a) displays the general training and validation losses, while sub-figure (b) displays the losses of the MR-CNN mask training and validation. The MR-CNN general loss is recorded as 0.1291 at the end of the training, and the MR-CNN mask loss is noted as 0.0450.

We evaluated the overlap between the annotated and the generated masks of the validation dataset using the Intersection Over Union (IoU) metric^29^. IoU is calculated using Eq. (10):

10 $$\begin{aligned} ({IOU})=\frac{Area\,of \,intersection\, of\, two\, masks }{Area\, of \,Union\, of\, two\, masks } \end{aligned}$$

A lower value of IoU indicates inadequate prediction (i.e. poor prediction), whereas a value of 1 represents an entirely accurate prediction. The validation process yielded an Average IoU of 0.9146, signifying that the model can be safely used.

### Imperceptibility measurement

The second part of the experiment was dedicated to the embedded process that uses the trained MR-CNN model. We tested the process on 10 images. After extracting patient information from the DICOM files and transforming them into QR codes, we applied the embedding method described above to conceal the QR codes (Message) in the Cover images to obtain Stego images as a first step. Then, we applied the extraction process to retrieve secret messages from the Stego images as a second step.

Table 2 shows the obtained results (the cover, message, Stego, and the Retrieved message) for a sample of 4 DICOM files. Based on visual inspection, it appears that there is no discernible disparity between the original (cover and message) and generated images (Stego and retrieved Qr code). However, we used the PSNR (peak signal to noise ratio) metric to evaluate the visual quality of the generated images and The CNN (Normalized Correlation Coefficient) metric to check the similarity between the cover mask and the stego mask obtained by the MR-CNN model, which affects the correctness of the extraction process.

**Table 2:** Comparative results showcasing cover, original QR code (message), stego image, and retrieved QR code.

	The cover	The message	The stego	The retrieved message
CT 1				
CT 2				
MRI 1				
MRI 2				

Furthermore, PSNR is calculated in decibels between two images using Eq. (12):

11 $$\begin{aligned} M S E= & {} \frac{\sum _{M, N}\left[ I_1(m, n)-I_2(m, n)\right] ^2}{M * N} \end{aligned}$$

12 $$\begin{aligned} P S N R= & {} 10 \log _{10}\left( \frac{R^2}{M S E}\right) \end{aligned}$$

where M and N are the numbers of rows and columns in the input images. R is the maximum fluctuation in the input image data type. Typical values for the PNSR for 16-bit data are between 30 and 80 dB.

Moreover, NCC is used in our case to measure the robustness of the model and estimate the difference between the cover mask and the Stego mask, NCC value adjacent to 1 implies that the two masks are similar. NCC formula is given in Eq. (13):

13 $$\begin{aligned} {N C C=\frac{\sum _{i=0}^m \sum _{j=0}^n (M C-\mu MC )(M S-\mu MS)}{\left( \sqrt{\sum _{i=0}^m(M C-\mu MC )^2}\right) \left( \sqrt{\sum _{i=0}^m (M S-\mu MS)^2}\right) }} \end{aligned}$$

where $\mu MC$ and $\mu MS$ are the mean pixel values of the cover mask and the Stego mask, respectively.

Table 3 presents the NCC and PSNR values for the tested DICOM images, along with their image sizes and scan types. The NCC values listed in the table fall within the range of 0.83–1 for all images, signifying that our MR-CNN models can predict identical masks from both the Cover and Stego images. This successful prediction enables the accurate detection of the insignificant area where concealment operations take place. The PNSR values depicted in Table 3, in case of the embedding parameters $\beta$ and $\alpha$ are set to 1000 and 0.02 respectively, are ranging between 70.45 and 82.38, indicating that our method effectively conceals sensitive patient information with a high level of imperceptibility.

**Table 3:** The NCC and PSNR values for the tested DICOM images.

Patient ID	1	2	3	4	5	6	7	8	9	10
Scan type	CT	CT	CT	CT	CT	MR	MR	MR	MR	MR
Image size (Pixel)	512 × 512	512 × 512	512 × 512	512 × 512	512 × 512	256 × 256	256 × 256	512 × 512	256 × 256	256 × 256
NCC	1.00	1.00	0.83	1.00	0.99	1.00	0.99	1.00	0.99	0.99
PSNR (dB)	116.07	113.69	115.53	116.57	116.02	107.47	110.3	117.4	110.3	110.33

To examine the effect of the $\beta$ coefficient on the final quality of the Stego, we measured the PSNR by variating the embedding parameter $\beta$. Table 4 shows the PSNR value obtained.

Table 4 shows the obtained average results of the MSE values between the Cover and Stego DICOM images of 20 patients for different b values. The average of the MSE values was between 2049.46 and 6152.98 for b = 500.While the minimum and maximum value of MSE was recorded for b = 1000 and b = 100, respectively, which prove that the largest value of b produces a high-quality Stego image. Table 4 shows the resultant average of PSNR values to compare between Cover and Stego images in decibel (dB). The average PSNR values ranged between 80.22 and 84.96 dB for b = 500. The minimum and maximum value of PSNR are 76.35 dB (patient 2 and b = 100) and 85.39 dB (patient 14 and b = 1000) respectively. Generally, PSNR higher values refer to the invisibility of higher quality.

**Table 4:** The average results of the MSE values between the Cover and Stego DICOM.

Scan type	$\beta$										
	10	100	200	300	400	500	600	700	800	900	1000
CT											
1	75.051	95.955	102.01	105.557	108.094	110.039	111.578	112.993	114.129	115.225	116.074
2	72.661	93.551	99.64	103.166	105.729	107.661	109.222	110.618	111.748	112.888	113.695
3	74.555	95.424	72.483	101.500	105.029	107.563	109.494	112.45	113.585	114.696	115.532
4	75.136	96.313	102.442	105.998	108.55	110.517	112.07	113.444	114.644	115.723	116.571
5	75.076	95.914	101.994	105.526	108.067	109.995	111.557	112.945	114.076	115.191	116.022
Averag	74.4958	95.4314	95.7138	104.3494	107.0938	109.155	110.7842	112.49	113.6364	114.7446	115.5788
MRI											
1	66.494	87.347	93.425	96.949	99.500	101.447	102.984	104.394	105.533	106.643	107.473
2	69.389	90.217	96.281	99.803	102.345	104.283	105.823	107.227	108.365	109.476	110.304
3	76.472	97.313	103.385	106.904	109.447	111.386	112.925	114.321	115.472	116.581	117.400
4	69.389	90.217	96.281	99.803	102.345	104.283	105.823	107.227	108.365	109.476	110.304
5	69.39	90.222	96.29	99.816	102.362	104.303	105.847	107.253	108.393	109.505	110.333
Averag	70.2268	91.0632	97.1324	100.655	103.1998	105.1404	106.6804	108.0844	109.2256	110.3362	111.1628

### Capacity and payload

Table 5 presents a detailed analysis of capacity and payload values for both CT (computed tomography) and MRI (magnetic resonance imaging) images across ten different patient IDs. These values provide crucial insights into the performance and efficiency of a data hiding technique when applied to medical images. It’s essential to consider these capacity and payload values when designing and implementing data hiding techniques for medical images, as they provide insights into the trade-off between data capacity and image quality in the context of medical data security. Capacity refers to the amount of secret data that can be embedded within the medical image while maintaining the image’s visual quality and integrity. For CT images, the capacity ranges from 0.48 to 0.72, with an average capacity of 0.54. This indicates that, on average, approximately 54% of the image can be utilized to hide secret data without significant degradation in image quality. For MRI images, the capacity varies from 0.49 to 0.63, with an average capacity of 0.58. MRI images show a slightly lower but still substantial capacity, with approximately 58% of the image available for data embedding. Payload refers to the amount of secret data that is successfully embedded within the image. It is a critical metric as it indicates how much data can be reliably hidden within the image. For CT images, the payload values range from 0.02 to 0.10, with an average payload of 0.05. This suggests that, on average, 5% of the image can be effectively used to conceal secret data. For MRI images, the payload varies between 0.02 and 0.10, with an average payload of 0.05, mirroring the payload results of CT images.

**Table 5:** The resultant capacity and payload.

	CT images					MRI images					
Patient id	1	2	3	4	5	6	7	8	9	10	Average
Capacity	0.5	0.54	0.50	0.48	0.49	0.63	0.61	0.54	0.72	0.58	0.54
Payload	0.031	0.037	0.031	0.032	0.031	0.09	0.10	0.02	0.08	0.10	0.05

### Robustness analysis

A critical aspect of our proposed data hiding technique for medical images is its robustness against various types of noise attacks. To evaluate the resilience of our method, we subjected the stego images to three common noise attacks: Gaussian noise, uniform noise, and salt and pepper noise. The robustness was assessed by retrieving the embedded QR code from the noise-affected stego images and measuring the similarity and visual quality through normalized cross-correlation (NCC) and peak signal-to-noise ratio (PSNR), respectively.

**Table 6:** The robustness evaluation results under the noise attacks.

Stego attack	Gaussian noise	Uniform noise	Salt and pepper noise
The retrieved QR code			
NCC	0.3188	0.3150	0.3193
PSNR (dB)	53.36	53.33	53.39

In Table 6, the NCC values obtained under Gaussian noise, uniform noise, and salt and pepper noise attacks were 0.3188, 0.3150, and 0.3193, respectively. These results indicate a moderate level of correlation between the original and retrieved QR codes post-attack, demonstrating the method’s capability to withstand noise perturbations to a certain extent. Moreover, the PSNR values remained above 53 dB across all noise types, suggesting that the visual quality of the stego images is preserved well above acceptable thresholds, even in the presence of noise. This is significant as it ensures that the diagnostic value of medical images is not compromised due to embedding and subsequent noise attacks.

In developing our data hiding approach for medical images based on the Mask-RCNN model, we meticulously balanced the trade-off between robustness, visual quality, and payload capacity. This delicate equilibrium ensures that while concealing data within the medical images, the method maintains resilience against various attacks, preserves high visual fidelity, and accommodates a significant payload for information embedding. By optimizing the embedding process and incorporating error correction coding techniques, we mitigate the risk of information loss and maintain the integrity of the stego images, even in the presence of noise or other forms of interference. Furthermore, careful selection of embedding parameters and compression algorithms allows us to strike an optimal balance between concealing capacity and visual imperceptibility, ensuring that the embedded data remains imperceptible to the human eye while maximizing the amount of information that can be securely hidden within the images. This careful consideration of trade-offs empowers our method to deliver robust and high-quality stego images suitable for secure transmission and storage of sensitive medical data.

### Comparison

Table 7 provides a comprehensive comparison between the proposed data hiding method and several other existing techniques, highlighting key parameters such as image size, embedding capacity, payload, and best PSNR (peak signal-to-noise ratio). Bozhidar and Borislav’s^8^ method employs a Nuclear Spin Generator on $336\times 336$ images, achieving a relatively high embedding capacity of 0.75 Bpp. It successfully hides 83,883 bits of data while maintaining a remarkable PSNR of 113.50, indicating good image quality preservation. Subhadip^20^ and his team utilize a combination of LSB (Least Significant Bit) and a shell matrix technique on $256\times 256$ images, resulting in a much higher embedding capacity of 3 Bpp. This approach allows the concealment of a substantial 786,432 bits of data. However, the PSNR of 48.42 indicates some loss in image quality compared to the previous method. Akshay^30^ and his collaborators employ deep learning on $128\times 128$ images, achieving an impressive embedding capacity of 24 Bpp. While the exact payload is not specified, this approach prioritizes data capacity over PSNR, which is lower at 37.55, indicating some visual quality degradation. Atique^31^ and his team also apply deep learning, but on $300\times 300$ images, resulting in an embedding capacity of 8 Bpp. They manage to conceal 89,910 bits of data with a PSNR of 36.58, indicating some trade-off between capacity and image quality. In contrast, the proposed method operates on $512\times 512$ images using deep learning and achieves a reasonable embedding capacity of 0.50 Bpp, which allows the concealment of 131,524 bits of data. Notably, it outperforms the other methods in terms of PSNR, attaining an impressive 115.53, signifying exceptional image quality preservation. In summary, the proposed method strikes a balance between embedding capacity and image quality, achieving a competitive capacity while maintaining outstanding PSNR, making it a promising choice for data hiding in medical images.

**Table 7:** Compare the proposed method with other methods.

Method	Image size	Technique	Embedding capacity (Bpp)	Payload (bits)	Best PSNR
Bozhidar and Borislav^8^	336 $\times$ 336	Nuclear spin generator	0.75	83,883	113.50
Subhadip et al.^20^	256 $\times$ 256	LSB and shell matrix	3	786,432	48.42
Akshay et al.^30^	128 $\times$ 128	Deep learning	24	–	37.55
Atique et al.^31^	300 $\times$ 300	Deep learning	8	89,910	36.58
Proposed method	512 $\times$ 512	Deep learning	0.50	131,524	**115.53**

Significant values are in [bold].

## Conclusion

In conclusion, this study has presented a novel approach to high-capacity data hiding for medical images based on the Mask-RCNN model. The Mask RCNN, as a deep learning segmentation model, is leveraged to identify medically less significant regions in DICOM images, where secret information, represented as a QR code, were embedded using DCT data hiding capabilities. The Mask RCNN model was fine-tuned and pretrained to consistently identify the same embedding regions in both original and Stego images, enabling reliable steganalysis. The necessity of this study is underscored by the ever-increasing reliance on digital medical imaging and the paramount importance of maintaining patient confidentiality in such a context.

The experimental outcomes, validated by well-recognized objective evaluation metrics such as peak signal-to-noise ratio (PSNR) and normalized cross-correlation (NCC), demonstrate our method’s superiority in achieving a delicate balance between embedding capacity, robustness against noise, and imperceptibility. The results demonstrated the effectiveness of the proposed technique in ensuring high capacity data embedding in medical images with low distortion. The introduced method has a minimum PSNR of 70 and a maximum PNSR of 115 which is large enough for the naked eye to not distinguish between the cover image and its stego image. Furthermore, this method demonstrates superior performance in comparison to its competitors.

The future work will delve into enhancing the robustness and scalability of our approach to accommodate diverse medical imaging modalities and data formats. Additionally, we aim to explore the integration of advanced encryption techniques to fortify the security of embedded data further. Furthermore, considering the dynamic nature of cybersecurity threats, continual evaluation and adaptation of our model will be essential to ensure its effectiveness in real-world clinical settings.

## Data Availability

nThe datasets generated and/or analysed during the current study are available from the corresponding authors upon request.
